# Deciphering the role of IGFBP5 in delaying fibrosis and sarcopenia in aging skeletal muscle: therapeutic implications and molecular mechanisms

**DOI:** 10.3389/fphar.2025.1557703

**Published:** 2025-03-12

**Authors:** Luze Shi, Zheci Ding, Jiwu Chen

**Affiliations:** Department of Sports Medicine, Shanghai General Hospital, Shanghai Jiao Tong University School of Medicine, Shanghai Jiao Tong University, Shanghai, China

**Keywords:** skeletal muscle fibroblasts, skeletal muscle fibrosis, skeletal muscle aging, fibrosis, sarcopenia

## Abstract

**Introduction:**

Sarcopenia is a condition characterized by the loss of muscle fibers and excessive deposition of extracellular matrix proteins. The interplay between muscle atrophy and fibrosis is a central feature of sarcopenia. While the mechanisms underlying skeletal muscle aging and fibrosis remain incompletely understood, cellular senescence has emerged as a key contributor. This study investigates the role of D-galactose (D-gal) in inducing fibroblasts senescence and skeletal muscle fibrosis, and aims to find the key regulator of the process to serve as a therapeutical target.

**Methods:**

To discover the role of D-gal in inducing cellular senescence and fibrosis, the senescence markers and the expression of fibrosis-related proteins were assessed after introducing D-gal among fibroblasts, and muscle strength and mass. The severity of muscle atrophy and fibrosis were also verified by using H&E staining and Masson trichrome staining after D-gal treatment via subcutaneous injection among mice. Subsequently, mRNA sequencing (RNA-seq) was performed and the differential expressed genes were identified between under D-gal or control treatment, to discover the key regulator of D-GAL-driven fibroblasts senescence and fibrosis. The role of the key regulator IGFBP5 were then validated in D-GAL treated IGFBP5-knockdown fibroblasts *in vitro* by analyzing the level of senescence and fibrosis-related markers. And the results were further confirmed *in vivo* in IGFBP5-knockdown SAMP8 mice with histological examinations.

**Results:**

D-gal treatment effectively induced cellular senescence and fibrosis in fibroblasts, as well as skeletal muscle atrophy, fibrosis and loss in muscle mass and function in mice. IGFBP5 was identified as a key regulator of D-GAL induced senescence and fibrosis among fibroblasts using RNA-seq. And further validation tests showed that IGFBP5-knockdown could alleviate D-GAL-induced fibroblast cellular senescence and fibrosis, as well as the severity of muscle atrophy and fibrosis in SAMP8 mice.

**Discussion:**

IGFBP5 emerging as a key regulator of D-GAL-induced fibroblast cellular senescence and fibrosis. The findings provide new insights into the molecular mechanisms underlying age-related skeletal muscle fibrosis and highlight IGFBP5 as a potential therapeutic target. Further research is needed to validate these findings and explore related clinical applications.

## Introduction

Sarcopenia, characterized by the loss of muscle mass and strength, and fibrosis, is a common health issues among the elderly, significantly impacting their mobility and overall health (Di Iorio et al., 2006; Tournadre et al., 2019). With the advent of the aging society, these concerns have garnered widespread attention. Skeletal muscle plays a crucial role in movement, metabolic balance, and heat generation (Argilés et al., 2016). Nevertheless, a range of abnormal health states, including long-term illnesses, malignancies, protracted infections, and the aging process, have the potential to upset the equilibrium between the synthesis and breakdown of muscle proteins. This disruption can subsequently result in the occurrence of muscle atrophy and fibrosis (Kirkendall and Garrett, 1998; Argilés et al., 2016). In the context of sarcopenia, fibrosis poses substantial detrimental effects on patients by escalating muscle stiffness and curtailing their physical activity levels (Argilés et al., 2016; Antar et al., 2023). The excessive accumulation of fibrous tissue can also interfere with the communication between muscle satellite cells and the surrounding cellular milieu, leading to a decline in their myogenic capabilities (Murphy et al., 2011; Serrano et al., 2011; Antar et al., 2023). Therefore, unveiling the mechanisms of fibrosis in aged muscle is fundamental for skeletal muscle health (Serrano et al., 2011; Liu et al., 2018).

Skeletal muscle fibroblasts and Fibro-Adipogenic Progenitors (FAPs) are both important for muscle repair and maintenance but have distinct roles(Molina et al., 2021; Chen et al., 2022; Chapman et al., 2016). Skeletal muscle fibroblasts primarily produce and remodel the extracellular matrix (ECM), supporting tissue structure and wound healing(Chapman et al., 2016; DeLeon-Pennell et al., 2020). In contrast, FAPs are specialized cells within skeletal muscle that aid regeneration by differentiating into adipocytes and fibroblasts in response to injury or disease(Molina, Fabre, and Dumont, 2021). FAPs secrete factors like IL-6 and WNT, which promote muscle repair and create a supportive environment for muscle stem cells (MuSCs) (Madaro et al., 2018; Riparini et al., 2022; Parker and Hamrick, 2021). Skeletal muscle fibroblasts maintain ECM and provide structural support(Gillies and Lieber, 2011), whereas FAPs have a dual role: they aid regeneration by supporting MuSCs but can also contribute to fibrosis or fat buildup in diseases like Duchenne Muscular Dystrophy (DMD) (Chen et al., 2022; Parker and Hamrick, 2021). Additionally, FAPs have broader differentiation potential, allowing them to become adipocytes or fibroblasts, influencing the balance between repair and fibrosis(Judson et al., 2017; Molina et al., 2021). Both cells contribute to muscle health and repair.

Regarding the various pathways involved in muscle fibrosis, oxidative stress and inflammation are significant for muscle atrophy and extracellular matrix (ECM) deposition, capable of activating numerous signal pathways, including the ubiquitin-proteasome system, autophagy-lysosome system, and mTOR (Nishikawa et al., 2021; Gambini and Stromsnes, 2022; Antar et al., 2023). The IGF (insulin-like growth factor) signaling pathway plays a crucial role in skeletal muscle fibrosis and sarcopenia (Clemmons, 2009; Ye et al., 2013; Frost and Lang, 2012; Forbes, Blyth, and Wit, 2020). Among them, IGF-1 is a key factor in this pathway (Hayashi et al., 2004). IGF-1 inhibits inflammation through the Ras/PI3K/IKK/NF-κB pathway, reducing pro-inflammatory cytokine production and promoting tissue repair 6. Chronic inflammation often leads to tissue atrophy due to prolonged cytokine exposure (e.g., TNF-α, IL-6), which disrupts cellular homeostasis. By suppressing NF-κB activation, IGF-1 mitigates inflammatory damage, indirectly preventing muscle or atrophy caused by persistent inflammation (Zhang et al., 2024; Feng et al., 2022; Stitt et al., 2004). Besides, IGF promotes muscle cell growth and differentiation by binding to the IGF-1 receptor and activating the downstream PI3K/Akt/mTOR signaling pathway, thus combating muscle atrophy (Yoshida and Delafontaine, 2020). There may also be an interaction between IGF-1 and TGF-β1, which together influence the process of skeletal muscle fibrosis (Danielpour and Song, 2006; Kjaer et al., 2006).

Insulin-like growth factor binding proteins (IGFBPs) are a group of proteins that bind to insulin-like growth factors (IGFs), finely regulating their biological activity, distribution, and mode of action (Kelley et al., 1996; Baxter, 2023). The IGFBP family includes at least seven different proteins (IGFBP-1 to IGFBP-7), which share similarities in structure and function but also possess some unique characteristics and roles (Kelley et al., 1996; Hwa et al., 1999; Allard and Duan, 2018). IGF binding protein 5 (IGFBP5), as a regulator of IGF-1, can influence the biological activity of IGF-1 and, consequently, the regenerative capacity of muscles (Hwa et al., 1999; Beattie et al., 2006). The biological functions of IGFBP5 remain a subject of debate in scientific research (Duan and Allard, 2020; Waters et al., 2022). Certain investigations propose that IGFBP5 could trigger senescence via the STAT3 pathway or pathways associated with P53. In contrast, other studies observe an increase in IGFBP5 levels in cells that have undergone senescence due to radiation or kinase inhibitor treatment (Alessio et al., 2024). Additionally, some reports associate reduced IGFBP5 expression with senescence (Nojima et al., 2022). The varied and sometimes conflicting biological functions ascribed to IGFBP5 might be due to its participation in multiple signaling pathways (Duan and Allard, 2020). However, the role of IGFBP5 in sarcopenia remains to be elucidated.

In the current study, a series of experiments were conducted *in vitro* and *in vivo* to undermine the mechanisms of skeletal muscle fibrosis under sarcopenic condition (Park et al., 2017; Lim and Frontera, 2023; Nojima et al., 2022). Relying on sequencing and verifications, IGFBP5 was noticed to be significantly upregulated in aged fibroblasts. Subsequently, we found that reducing the expression of IGFBP5 partially alleviated fibrosis in sarcopenic muscle by moderately potentiating the effects of IGF-1, providing clue to the development of novel anti-fibrosis therapies in sarcopenia.

## Materials and methods

### Cell culture and induction

Mouse skeletal muscle fibroblast cells (NOR-10) were purchased from the Shanghai Zhong Qiao Xin Zhou Biotechnology Co., Cells were cultured in Dulbecco’s modified Eagle medium supplemented with 10% fetal bovine serum and 1% penicillin/streptomycin. Cells were maintained in a humidified incubator at 37°C and 5% CO2 atmosphere. FAPs were isolated from skeletal muscle tissues according to the previous study and cultured in DMEM supplemented with 10% FBS, 1% penicillin-streptomycin, and 1% L-glutamine (Kang et al., 2024). For the induction of senescence from fibroblast and FAPs, cells were incubated in a D-gal concentration of 20 mg/mL for 3 days, while the negative control (NC) group was treated with an equal amount of PBS.

### siRNA structure and design and transfection

Small interfering RNA (siRNA) molecules were designed to specifically target the mRNA of the gene, IGFBP5, to induce RNA interference (RNAi) and achieve gene silencing. The sequences of the siRNA were designed based on the mRNA sequence of IGFBP5 (GenBank Accession No. NM_010518). Cells were seeded in 24-well plates. When cells reached 30%–50% confluence, siRNA was transfected using Lipofectamine 2000. siRNA and Lipofectamine 2000 were diluted in Opti-MEM I, mixed, and incubated for 20 min at room temperature. The complex was added to the cells, incubated at 37°C in 5% CO_2_, and after 4–6 h, replaced with complete medium containing 10% FBS. Cells were harvested 48 h later for analysis.

### Senescence-associated β-galactosidase(SA-β-gal) staining

The protocol was consistent with the previous study (Shahini et al., 2021), that n = 3 biological replicates were used. Digital camera was used to capture images of the stained cells. ImageJ (Version 1.54 m) was used to count the number of blue-stained senescent cells and the total number of cells in each image.

### Transcriptome sequencing (RNA sequencing) and bioinformatic analysis

Raw data was obtained with Feature Extraction software 10.7 and normalized (GSE277119). For fibroblasts induced by D-gal and control group samples (n = 3 in each group), sequencing libraries were generated using NEBNext^®^ Multiplex Small RNA Library Prep Set for Illumina^®^ (NEB, USA). Raw sequencing reads were processed using FastQC (version 0.11.9) to assess the quality of the sequencing data. Low-quality reads (Phred score <20) were trimmed using Trimmomatic (version 0.39). High-quality reads were retained for further analysis. Genes were considered differentially expressed if they met the following criteria: an adjusted p-value (FDR) < 0.05 and a log2 fold change (log2FC) ≥ 1 or ≤ −1. GO (Gene Ontology) and KEGG (Kyoto Encyclopedia of Genes and Genomes) pathway enrichment analysis were performed as the protocol according to the previous study (Sun et al., 2018).

### Animals

Healthy male C57BL/6 mice, 10 in total, 6–8 weeks old, with body weights ranging from 20 to 24 g, purchased from Cyagen Biosciences. Mice was randomly divided into experimental and control groups (n = 5), with the experimental group mice receiving D-gal via subcutaneous injection at a dose of 200 mg/kg/d for 8 consecutive weeks. The control group is injected with an equivalent amount of normal saline. The SAMP8 (senescence-accelerated mouse-prone 8) model was chosen for its accelerated aging phenotype, which mimics age-related fibrogenic processes in skeletal muscle. SAMR1 (senescence-accelerated mouse-resistant 1) was used as a control. Healthy male SAMP8, 10 in total, and SAMR1, 5 in total, 24 weeks old, with body weights ranging from 42 to 45 g, purchased from Hangzhou Ziyuan Experimental Anmial Technology Co. Mice was randomly divided into experimental and control groups (n = 5), with the experimental group mice receiving siRNA dissolved in normal saline via tail vein injection at a dose of 100 umol/ml twice a week for 4 consecutive weeks. The control group and the SAMR1 group are injected with an equivalent amount of normal saline. The mice were housed separately, and had sufficient space to meet the growth and behavioral needs of the animals, provided with feed and distilled water. Bedding was kept clean with good air circulation. 1 day after the last injection, mice were placed sacrificed with carbon dioxide,and the CO2 flow was 30% vessel volume per minute to ensure that the animal gradually became consciousness and eventually died before reaching a concentration that could cause pain. The lower limbs of the mouse were carefully amputated, and the muscles (gastrocnemius, tibialis anterior, quadriceps) were dissected away from the bone and surrounding tissues. Department of Shanghai Chedun Experimental Animal Ethics Committee provided full approval for this research (AD2024092).

### Western blot

Western blot (WB) was performed following the procedures in a previous publication(n = 3) (Zhang et al., 2023), with primary antibodies identified by the following catalog numbers: P16(10883-1-AP), P53(10442-1-AP), IGFBP5(55205-1-AP), COL-1(14695-1-AP), α-SMA(14395-1-AP).

### PCR

The PCR was performed according to the protocols established in a previous study (Mollica, 2010).

### HE, MASSON and immunofluorescence staining

All staining protocols were adhered to as described in previous studies(n = 3) (Wang et al., 2017; Van De Vlekkert et al., 2020; Esper et al., 2023), with primary antibodies identified by the following catalog numbers: IGFBP5(55205-1-AP), α-SMA(14395-1-AP), IGF-1(28530-1-AP), TGF-β(26155-1-AP). Fluorescence intensity was measured using a fluorescence microscope and normalized to control.

### Statistical analysis

All data are presented as mean ± standard deviation (SD). GraphPad Prism 9.4.1 software (GraphPad, CA, USA) was used for statistical analysis and image construction. For comparisons between two groups, Student’s t-test and Paired Samples t-test was used. For comparisons among multiple groups, one-way ANOVA was employed, followed by *post hoc* Tukey’s test for pairwise comparisons. All statistical tests were two-tailed, and p-values less than 0.05 were considered statistically significant.

## Results

### D-gal-induced skeletal muscle fibrosis characteristics

**FIGURE 1:**
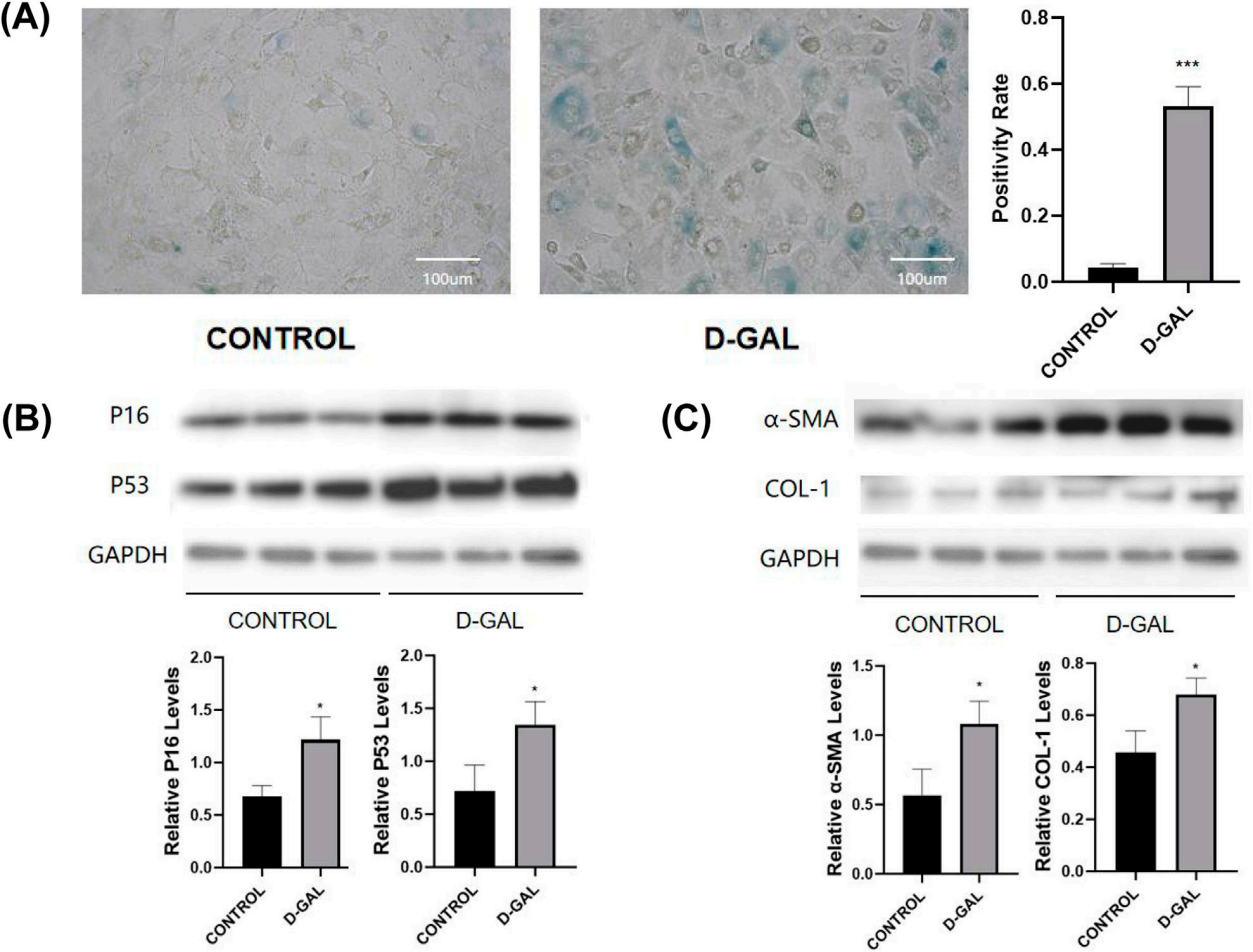
**(A)** SA-β-gal staining of NOR-10 (CONTROL vs. D-GAL) and the statistical analysis **(B)** Western blot of senescence markers (P16, P53) and the statistical analysis **(C)** Western blot of fibrosis-related markers (α-SMA, COL-1) and the statistical analysis (compared to control group, *p < 0.05, **p < 0.01, ***p < 0.001).

D-gal is a chemical that commonly induces cellular senescence (Azman and Zakaria, 2019). It leads to mitochondrial damage and a decline in energy metabolism, which are associated with aging (Parameshwaran et al., 2010). The first, NOR-10 fibroblasts were treated with D-gal, resulting in significant increases in the protein levels of senescence markers p16 and p53, and SA-β-gal staining confirmed cellular aging (Figures 1A, B). This confirms the successful establishment of an aging model in mouse skeletal muscle fibroblasts post D-gal induction. Western blot of fibrosis-related markers (α-SMA, COL-1) were then detected (Figure 1C). Not surprisingly, a significant elevation was observed after D-gal induction.

**FIGURE 2:**
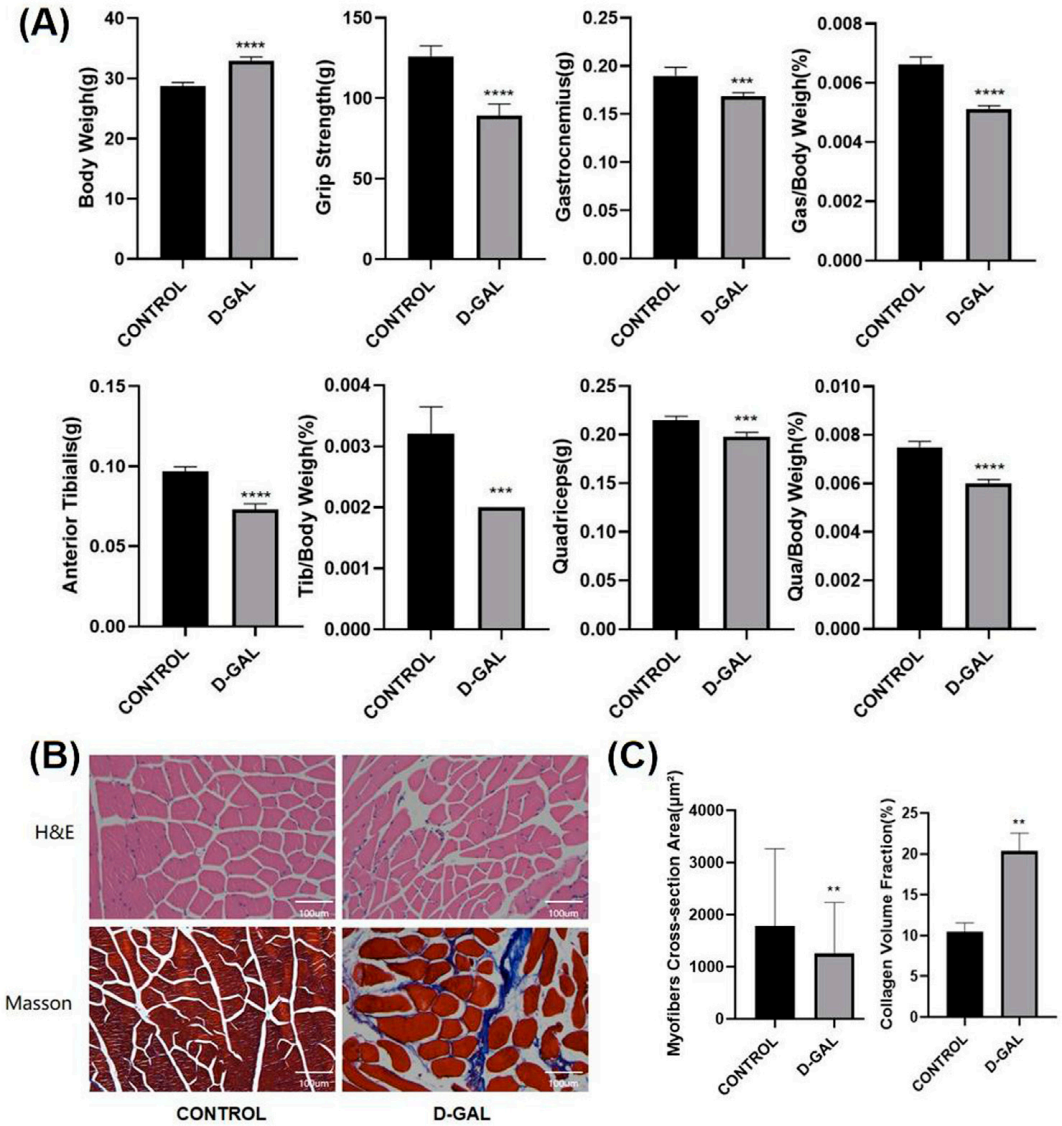
**(A)** Body weight, muscle strength, and the weight of lower limb skeletal muscles (gastrocnemius, tibialis anterior, quadriceps) and their percentage of the body weight of C57BL/6 mice(CONTROL vs. D-GAL) **(B)** The HE and Masson staining of limb skeletal muscle(CONTROL vs. D-GAL) **(C)** the statistical analysis of, myofiber cross sectional area (CSA) and collagen volume fraction (CVF) (compared to control group, *p < 0.05, **p < 0.01, ***p < 0.001, **** *p* < 0.0001).

According to the research methods in previous articles, D-gal is also widely used to induce skeletal muscle aging (Tian et al., 2022). After 8 weeks of D-gal injection, comparisons were made in terms of body weight, muscle strength, and the weight of lower limb muscles (gastrocnemius, tibialis anterior, quadriceps) and their percentage of the body weight (Figure 2A). The results demonstrated that the D-gal induced group had a significant decrease in muscle strength and slight decline in the weight of individual lower limb muscles. HE and Masson showed a significant reduction in fiber cross-sectional area with, on the other hand, a noticeable increase in ECM in the D-gal induced group (Figures 2B, C).

### Identifying IGFBP5 in D-gal-induced skeletal muscle sequencing analysis

**FIGURE 3:**
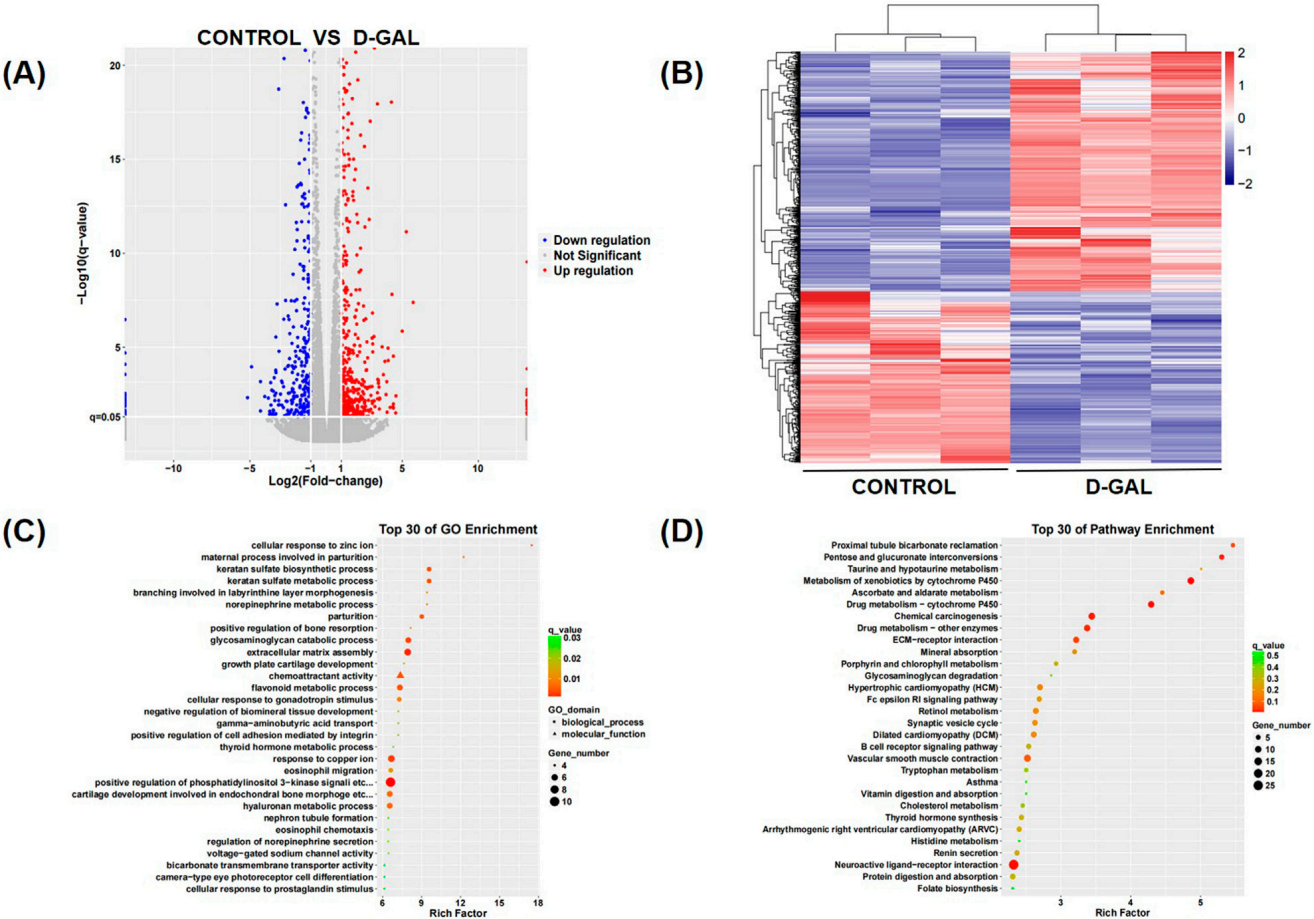
**(A)** The heatmap of differential genes of the sequencing results **(B)** The volcano plot of differential genes of the sequencing results **(C)** The GO enrichment of differential genes of the sequencing results **(D)** The KEGG enrichment of differential genes of the sequencing results.

To explore the mechanisms underlying the fibrosis of skeletal muscle during its aging process, we performed sequencing on skeletal muscle fibroblasts that had been induced to aging. Compared to the sequencing results of the control group, there were significant differences in mRNA expression (Figures 3A, B). Enrichment analysis was conducted using Gene Ontology (GO) and Kyoto Encyclopedia of Genes and Genomes (KEGG) pathways, and pathways related to skeletal muscle fibrosis were identified as being of particular interest in cellular processes, regulation of biological processes, and metabolism, such as transporter activity, translation regulator activity, ECM-receptor interactions and cell growth and death (Figures 3C, D). Differential gene expression was selected by data processing, including both upregulated and downregulated genes (Supplementary Figures 1A–D). By further analyzing the gene enrichment results from KEGG and GO, and conducting a search and study of relevant literature and currently published research articles, the IGFBP5 gene has been identified. IGFBP5 was identified as a key candidate gene of differentially expressed genes (DEGs) and the expression of it was significantly upregulated compared to controls (log2 fold change > 2, p < 0.01).

### IGFBP5 is highly expressed in the senescence

The protein level in cells induced by D-gal of insulin-like growth factor binding protein 5 (IGFBP5) has also exhibited a noticeable elevation, suggesting its potential role in the fibrotic process (Supplementary Figure 2). Additionally, we performed PCR validation using FAPs cells. In FAPs induced by D-gal, the markers of senescence, fibrosis, and adipogenesis were all increased, along with an elevation in IGFBP5 (Supplementary Figure 3). This indicates that within the skeletal muscle aging model, the skeletal muscle not only shows characteristics of fibrosis but also an upregulation in the expression of IGFBP5, aligning with the sequencing results. Immunofluorescence staining in the D-gal-induced aging animal model has revealed a significant increase in the expression of α-SMA. Furthermore, IGFBP5 has shown a more pronounced and widespread distribution in skeletal muscle compared to the control group, indicating a possible association between IGFBP5 expression and the aging process in skeletal muscle (Supplementary Figure 4). These findings suggest that the D-gal-induced aging model is associated with a notable increase in skeletal muscle fibrosis and a high expression of IGFBP5, which may play a role in the fibrotic response to aging.

### Knockout of IGFBP5 alleviates fibrosis in the aging model

**FIGURE 4:**
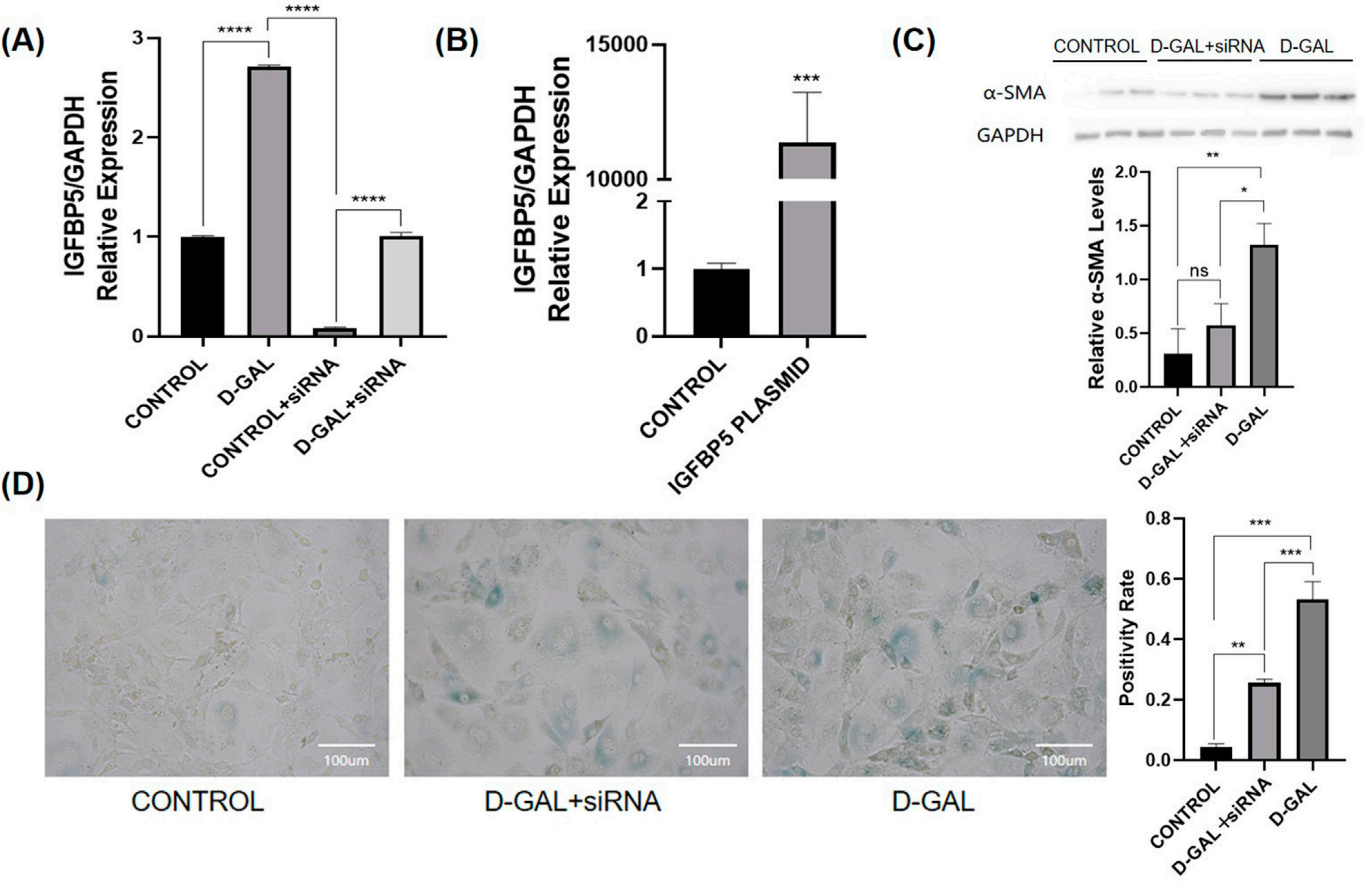
**(A)** The PCR of IGFGP5 (CONTROL vs. D-GAL vs. CONTROL + siRNA vs. D-GAL + siRNA); **(B)** The PCR of IGFBP5 (CONTROL vs. PLASMID) **(C)** Western blot of α-SMA and the statistical analysis **(D)** SA-β-gal staining of NOR-10 and the statistical analysis (CONTROL vs. D-GAL + siRNA vs. D-GAL) (compared to control group, *p < 0.05, **p < 0.01, ***p < 0.001, **** *p* < 0.0001).

**FIGURE 5:**
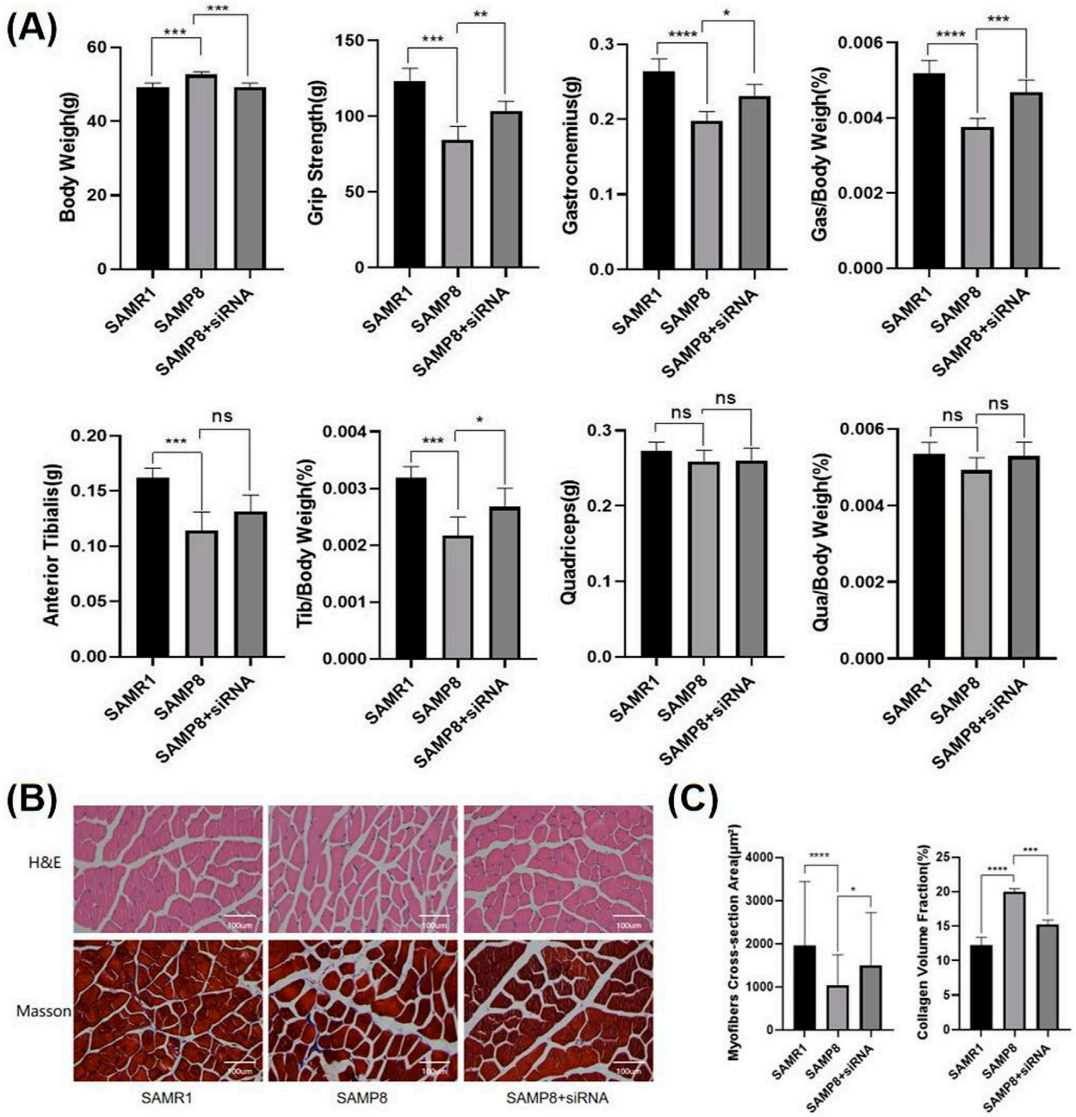
**(A)** Body weight, muscle strength, and the weight of lower limb skeletal muscles (gastrocnemius, tibialis anterior, quadriceps) and their percentage of the body weight of mice(SAMR1 vs. SAMP8 vs. SAMP8+siRNA) **(B)** The HE and Masson staining of limb skeletal muscle(SAMR1 vs. SAMP8 vs. SAMP8+siRNA) **(C)** the statistical analysis of myofiber CSA and CVF (compared to control group, *p < 0.05, **p < 0.01, ***p < 0.001, **** p < 0.0001).

To investigate the specific mechanisms of action of IGFBP5 at the cellular level, siRNA and plasmids were selected (Figures 4A, B). In NOR-10 cells induced by D-gal, protein level analysis revealed that the fibrosis level in fibroblasts decreased after the knockout of IGFBP5 (Figure 4C). Additionally, the senescence of cells with IGFBP5 knockout was significantly improved (Figure 4D). Moreover, SAMP8 mice were selected for the study. 24-week-old mice was chosen for the experiment, administering siRNA via tail vein injection for 4 weeks to knock down the expression of IGFBP5 in aging mice. At the end of the modeling, consistent with the previous text, the mice’s body weight, muscle strength, and the weight of lower limb muscles (gastrocnemius, tibialis anterior, quadriceps) and their percentage related to mice weight were assessed (Figure 5A). The results demonstrated that in the IGFBP5 knockdown group, there was a moderate decrease in body weight, a significant improvement in muscle strength, and a noticeable increase in the weight of the lower limb muscles. The percentage was not as significantly improved, but there was a general upward trend. Tissue section staining with HE and Masson also showed that the degree of fibrosis in skeletal muscle was improved in mice with IGFBP5 knockdown. This was manifested as a significantly larger cross-sectional area of muscle fibers in the siRNA group compared to aging mice, improved gaps between muscle fibers, and relatively less connective tissue compared to aging mice, although it did not reach the condition of normal adult mice (Figures 5B, C).

### IGFBP5 regulates skeletal muscle fibrosis through IGF-1

**FIGURE 6:**
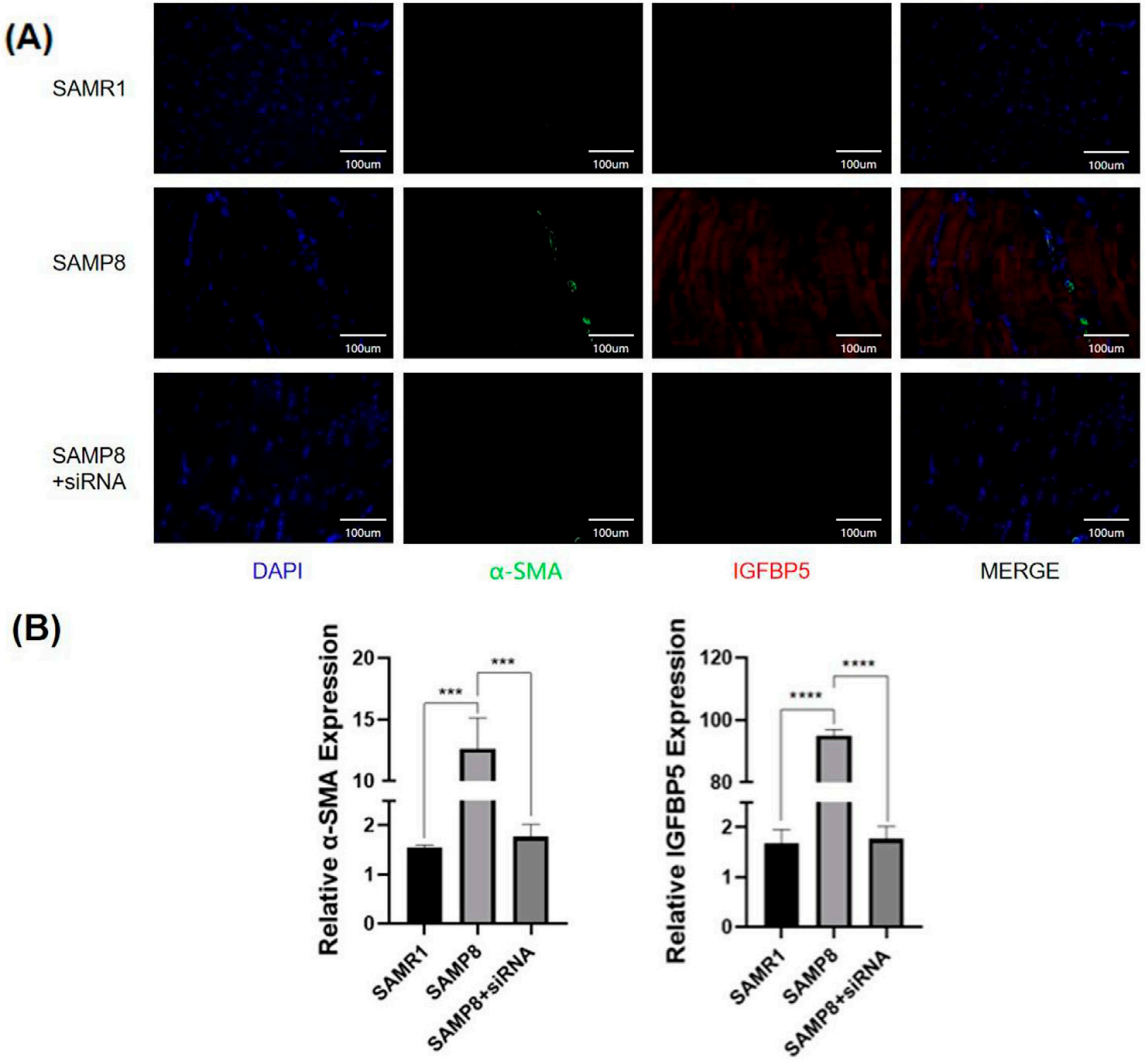
**(A)**The immunofluorescence staining of skeletal muscle ( α-SMA , IGFBP5 ) (SAMR1 vs. SAMP8 vs. SAMP8+siRNA) **(B)** Statistical analysis of the positive expression (compared to control group, *p < 0.05, **p < 0.01, ***p < 0.001, **** p < 0.0001).

Immunofluorescence staining of muscle tissue from aging mice and mice with IGFBP5 knockout revealed that the expression of the fibrosis marker α-SMA was significantly reduced in mice with IGFBP5 knockout (Figure 6). Notably, IGFBP5 expression was also substantially decreased (Figure 6). This indicates that IGFBP5 can indeed alleviate skeletal muscle fibrosis. IGF-1 can affect TGF-β1 activity, a cytokine linked to fibrosis. It also regulates ECM buildup, key in muscle fibrosis. IGF-1 is the main route for IGFBP5’s effects, with IGFBP5 impacting processes both with and without IGF-1. The signaling pathway involves a complex network of genes. This study focuses specifically on investigating whether IGFBP5 can regulate skeletal muscle fibrosis in an IGF-1-dependent manner, without delving into the deeper mechanistic aspects of its action. To verify this, immunofluorescence staining of skeletal muscle tissue was performed again, and it was found that in SAMP8 mice with IGFBP5 knockout, the expression of IGF-1 was increased compared to aging SAMP8 mice (Supplementary Figure 5). TGF- β staining was also performed, and TGF- β expression was reduced in the IGFBP5 knockout mice (Supplementary Figure 5). This suggests that IGFBP5 may modulate the process of skeletal muscle fibrosis by mediating interactions with both IGF-1 and TGF-β pathways.

## Discussion

The interplay between muscle atrophy and fibrosis is a central aspect of sarcopenia (Boccardi, 2024). While muscle atrophy involves the loss of muscle fibers, fibrosis refers to the excessive deposition of extracellular matrix (ECM) proteins, particularly collagen, which leads to muscle stiffness and a reduction in physical activity levels (Bonaldo and Sandri, 2013; Sakuma et al., 2014; Mahdy, 2019). This combination not only impairs mobility but also disrupts the communication between muscle satellite cells and their environment, thereby compromising the muscle’s regenerative capacity (Blau et al., 2015; Hong et al., 2022).

Skeletal muscle fibroblasts are essential cells within skeletal muscle that play a multifaceted role in maintaining muscle structure, function, and homeostasis (Chapman et al., 2016). These cells are primarily responsible for the synthesis and secretion of extracellular matrix (ECM) components, such as collagen, elastin, and glycosaminoglycans, which provide mechanical support and structural integrity to muscle fibers (Plikus et al., 2021; Chapman et al., 2016). In addition to their structural role, skeletal muscle fibroblasts are crucial for tissue repair and regeneration following injury (Tidball, 2011; Younesi et al., 2024). Upon activation, these fibroblasts can differentiate into myofibroblasts, which express contractile proteins like α-smooth muscle actin (α-SMA) and contribute to the formation of scar tissue (Younesi et al., 2024; Hall et al., 2023; Gibb et al., 2020). However, excessive or prolonged activation of myofibroblasts can lead to pathological fibrosis (Schuster et al., 2023; Younesi et al., 2024). Moreover, fibroblasts play a significant role in regulating inflammation and immune responses (Davidson et al., 2021; Chapman et al., 2016). Additionally, skeletal muscle fibroblasts interact closely with muscle cells, influencing their growth, differentiation, and contractile function through the secretion of growth factors like IGF-1 and by providing mechanical signals (Chapman et al., 2016; Murphy et al., 2011; Abdel-Raouf et al., 2021). Their functions extend beyond structural support to include critical roles in immune regulation and cellular communication, highlighting their importance in both physiological and pathological contexts (Chapman et al., 2016).

Fibro-adipogenic progenitors (FAPs) are mesenchymal stromal cells residing in skeletal muscle interstitium, playing dual roles in muscle homeostasis, regeneration, and pathology (Joe et al., 2010; Uezumi et al., 2010). Following muscle injury, FAPs rapidly activate, proliferate, and transiently expand to orchestrate regeneration, which promote muscle satellite cell (MuSCs) proliferation and differentiation into myofibers (Heredia et al., 2013). This post-injury pro-regenerative response was tightly regulated by inflammatory signals such as TNF-α, while anti-inflammatory cytokines such as IL-4 and IL-13 later induce FAPs apoptosis, preventing excessive extracellular matrix (ECM) deposition (Lemos et al., 2015). Dysregulation of this balance leads to pathological outcomes, where FAPs underwent fibro-adipogenic differentiation, replacing functional muscle tissue and impairing contractility (Natarajan et al., 2010).

Notably, FAPs exhibit microenvironment-dependent plasticity. While their crosstalk with MuSCs is essential for repair, aberrant signaling such as TGF-β overactivation shifts FAPs toward a profibrotic state (Contreras et al., 2019). Recent studies highlight their dual nature—indispensable for regeneration yet potential drivers of degenerative diseases. Therefore, FAPs are pivotal regulators of skeletal muscle dynamics, balancing regenerative support with risks of pathological tissue remodeling, making them critical targets for muscle disease therapies. However, their complex mechanisms of action and interactions with numerous other cellular pathways make it challenging to elucidate a singular mechanism. In this study, we focus on skeletal muscle fibroblasts as the primary cell type for investigation, although FAPs are also employed for some key validations.

D-gal is a widely used chemical to induce cellular senescence (Azman and Zakaria, 2019). Cells undergoing senescence induced by D-gal exhibit mitochondrial structural damage and a decline in energy metabolism, which are highly related to cellular aging studies have confirmed that D-gal can induce senescence, fibrosis, and redox imbalance in skeletal muscle fibroblasts (Wu et al., 2022; Ma et al., 2024). Our results confirm that D-gal effectively induces cellular senescence and skeletal muscle fibrosis in both cellular and animal models. The increase in senescence markers and fibrosis-related proteins, along with the observed decline in muscle strength and mass, are consistent with previous studies that highlight the role of D-gal in modeling aging-associated pathologies. The observed lethargy and reduction in muscle fiber cross-sectional area further validate the model’s relevance to sarcopenia research.

The sequencing analysis conducted in our study has unveiled substantial alterations in mRNA expression, pinpointing IGFBP5 as a potential regulator of skeletal muscle fibrosis. This discovery associates with existing literature, which posits that IGFBP5 plays a complex and multifaceted role in cellular processes, particularly in the realms of cell growth and metabolism regulation. The overexpression of IGFBP5 in senescent skeletal muscle fibroblasts, coupled with its association with elevated markers of fibrosis, highlights its potential as a therapeutic target for interventions aimed at combating fibrosis. IGFBP5 is highly conserved in evolution compared to other IGFBP proteins and possesses a variety of biological activities (Duan and Allard, 2020). Existing research has demonstrated that IGFBP5 can play a role in the regulation of cell growth and metabolism by mediating the IGF1 signaling pathway (Ding et al., 2016). However, in addition to its function through the IGF signaling pathway, IGFBP5 also has IGF-independent activity, which adds to the complexity of its regulation of cellular behavior (Duan and Allard, 2020; Dittmer, 2022). We further investigate whether IGFBP5 can affect the fibrotic phenotype of skeletal muscle in an IGF-1-dependent manner.

The intricate role of IGFBP5 extends beyond its interaction with insulin-like growth factors (IGFs) (Beattie et al., 2006). It is known to modulate IGF bioavailability by binding to IGFs, thereby influencing the activity of the IGF signaling pathway (Clemmons, 2016). This pathway is crucial for various physiological processes, including muscle growth and repair. Based on the provided search results, there is no direct evidence discussing the regulation of IGFBP5 expression in fibroblasts and FAPs. However, IGFBP5 were found to be associated with fibrotic pathways in other tissue, suggesting the possibility that the expression of IGFBP5 could also be regulated under muscle pathologies (Contreras et al., 2021; Sorokina et al., 2024; Babaeijandaghi et al., 2023; Li et al., 2025). This study aims to investigate the role of IGFBP5 in the fibrosis of aging skeletal muscle. In the *in vivo* experiments conducted in this paper, it was found that in SAMP8 mice with knockdown of IGFBP5, there was a noticeable improvement in muscle strength, and both the weight and cross-sectional area of the skeletal muscles were improved to some extent. This indicates that the knockdown of IGFBP5 can partially ameliorate the quality of aging skeletal muscle. Staining of the skeletal muscles also showed a reduction in the degree of fibrosis, and the expression of IGF-1 increased to some extent after the knockdown of IGFBP5. This suggests that IGFBP5 can act through the regulation of IGF-1 in the fibrosis of aging skeletal muscle. In previous research related to skeletal muscle, there is literature supporting that IGFBP5 can function as a growth factor regulating skeletal muscle growth and also plays a role in disuse atrophy of skeletal muscle.

Mice and humans share a high degree of similarity in genetic mechanisms and physiological characteristics, which is why mouse models are widely used in medical research on human aging (Breschi et al., 2017). One of the most commonly used strains is the C57BL/6J mouse; almost all biological markers can detect aging changes in mice aged 18–24 months, making it a frequently used model for natural aging (Wu et al., 2024). The D-gal-induced aging model involves the continuous injection of D-gal into animals over a certain period, leading to an increase in galactose concentration within cells (Wang et al., 2023). Under the catalysis of aldose reductase, galactose is reduced to galactitol, which cannot be further metabolized by cells and accumulates, affecting osmotic pressure, causing cell swelling and dysfunction, ultimately leading to aging (Azman and Zakaria, 2019; Azman et al., 2021). Initially used to establish cataract models, this model has been developed through continuous research, and its various biochemical and physiological indicators are similar to natural aging, making it widely used today (Azman and Zakaria, 2019). The senescence-accelerated mouse (SAM) is a kind of premature aging model mouse, including two strains: SAMP (senescence accelerated-prone mouse) and SAMR (senescence accelerated resistant mouse) (Chiba et al., 2009; Takeda, 2009). SAMP exhibits rapid aging characteristics after a normal growth period (Takeda, 2009). SAMP8, a sub-strain of SAMP, is currently recognized as an ideal model for natural aging and dementia (Butterfield and Poon, 2005; Liu et al., 2020). In this article, the D-gal aging model and the SAMP8 premature aging mouse model were selected for their short modeling time and simple operation. Many pathways and targets related to skeletal muscle have been identified in these two models, such as the Wnt/β-catenin signaling pathway and its downstream cascade (Rudolf et al., 2016), the AMPK/TGF-β/SMAD axis (Zhong et al., 2024), and important skeletal muscle-related pathways, as well as targets related to skeletal muscle fibrosis and atrophy, such as CILP2 and TRIM16 (Deng et al., 2024; Guo et al., 2024). This study found that the IGFBP5 target may regulate the progression of fibrosis and sarcopenia in aging skeletal muscle through the IGF-1 pathway.

In this study, we also observed a seesaw effect between IGF-1 and TGF-β. The role of TGF-β in skeletal muscle fibrosis is undoubted. In skeletal muscle fibrotic pathologies, TGF-β1 is highly expressed and plays a key role in the development of skeletal muscle fibrosis (Ismaeel et al., 2019; Budi et al., 2021). It can promote the expression of extracellular matrix (ECM) components such as collagen and fibronectin and inhibit ECM degradation, playing a significant role in cell morphogenesis, proliferation, and differentiation processes (Roberts et al., 1992; Akhurst, 2004; Massagué and Sheppard, 2023). The activation of the TGF-β signaling pathway leads to pathological fibrosis (Meng et al., 2016). IGF-1 also plays a very important positive role in the growth and development of skeletal muscle, can delay various pathological muscle atrophies, and maintain and promote the growth and survival of the nervous system (Yoshida and Delafontaine, 2020; Ahmad et al., 2020). The decline in skeletal muscle mass and strength (sarcopenia) is also related to the reduced activity of the IGF-1/Akt/mTOR signaling pathway (Feng, 2010; López-Caamal et al., 2012). Both TGF-β and IGF-1 are important factors in skeletal muscle, and our research suggests that IGFBP5 may affect skeletal muscle aging and fibrosis by regulating the dynamic balance between TGF-β and IGF-1 through the expression of regulatory factors.

While our study focuses on the role of IGFBP5 in skeletal muscle fibrosis and sarcopenia, its involvement in fibrosis extends to multiple tissues and disease states, highlighting its potential as a therapeutic target. IGFBP5 is upregulated in idiopathic pulmonary fibrosis (IPF) and contributes to fibroblast activation and ECM remodeling. Elevated IGFBP5 levels in bronchoalveolar lavage fluid correlate with disease severity, suggesting its potential as a biomarker (Sureshbabu et al., 2011). In heart failure and myocardial infarction, IGFBP5 plays a dual role in fibrosis and repair, and also supports angiogenesis and cardiomyocyte survival under stress, highlighting its context-dependent roles (Zhu et al., 2024). IGFBP5 promotes fibroblast-to-myofibroblast transition and collagen synthesis, and interacts with ECM components (e.g., collagen I, III) to stabilize fibrotic lesions (Sureshbabu et al., 2009). Moreover, IGFBP5 has been shown to act independently of IGF-1 in other cell and disease models, indicating a complexity that warrants additional research (Duan and Allard, 2020; Dittmer, 2022). The interplay between TGF-β and IGF1 is not confined to a single pathway and requires further exploration. By elucidating the broader role of IGFBP5 in fibrotic disorders, our study not only advances understanding of its mechanisms in sarcopenia but also highlights its relevance across multiple diseases. This positions IGFBP5 as a promising target for anti-fibrotic therapies, with potential applications in pulmonary, cardiac, renal, hepatic, and dermal fibrosis.

In the present study, several limitations should be acknowledged. Firstly, naturally aged mice were not utilized, which may limit the direct relevance of the findings to natural aging processes. The relationships among SAMP8, SMAR1, and SAMP8 with siRNA require further investigation to elucidate their interactions and potential synergistic effects. Furthermore, in in vivo models, the injection of siRNA may potentially impact other cells within the skeletal muscle, not just limited to NOR-10. This necessitates further validation in subsequent studies. Additionally, conditional knockout mice were not employed, which could have provided more precise insights into gene-specific functions and their roles in the studied processes. Future research should address these limitations to enhance the robustness and applicability of the findings, and explore tissue-specific IGFBP5 regulation and its interplay with other fibrogenic factors to develop precision therapies.

These findings offer new insights into understanding age-related skeletal muscle fibrosis and provide potential molecular targets for the development of therapeutic strategies aimed at skeletal muscle fibrosis. By modulating the expression or activity of IGFBP5, it may be possible to slow down or reverse skeletal muscle fibrosis, thereby improving muscle function and quality of life in the elderly.

## Data Availability

The datasets presented in this study can be found in online repositories. The names of the repository/repositories and accession number(s) can be found below: https://www.ncbi.nlm.nih.gov/geo/, GSE285869.

## Supplementary material

SUPPLEMENTARY FIGURE S1 Images of related-IGFBP5.

SUPPLEMENTARY FIGURE S2 Western blot of IGFBP5 and the statistical analysis (compared to control group, *p < 0.05, **p < 0.01, ***p < 0.001, **** p < 0.0001).

SUPPLEMENTARY FIGURE S3 The PCR of P16,P21,PPARG,COL-1 and IGFBP5 (compared to control group, *p < 0.05, **p < 0.01, ***p < 0.001).

SUPPLEMENTARY FIGURE S4 The immunofluorescence staining of skeletal muscle (α-SMA, IGFBP5) (CONTROL vs. D-GAL).

SUPPLEMENTARY FIGURE S5 The immunofluorescence staining of skeletal muscle (IGF-1, TGF-β) (SAMP8 vs. SAMP8+siRNA).
