# Supplementary figures and images for: Deciphering the role of IGFBP5 in delaying fibrosis and sarcopenia in aging skeletal muscle: therapeutic implications and molecular mechanisms
Source: Front Pharmacol. 2025 Mar 12;16:1557703. doi: 10.3389/fphar.2025.1557703 (PMC11937025; doi:10.3389/fphar.2025.1557703)

## Slide 1
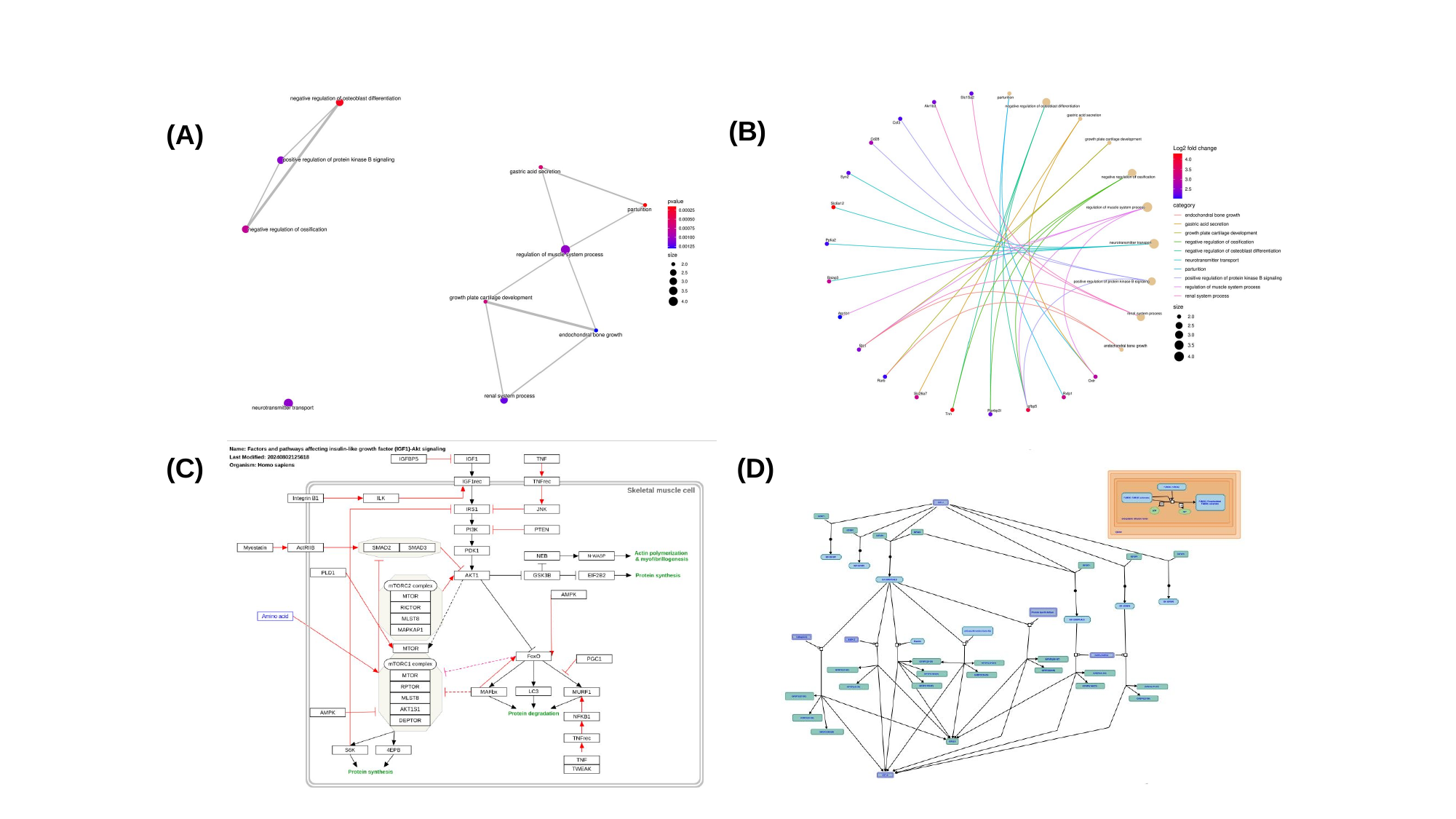

(B)
(A)
(C)
(D)

## Slide 2
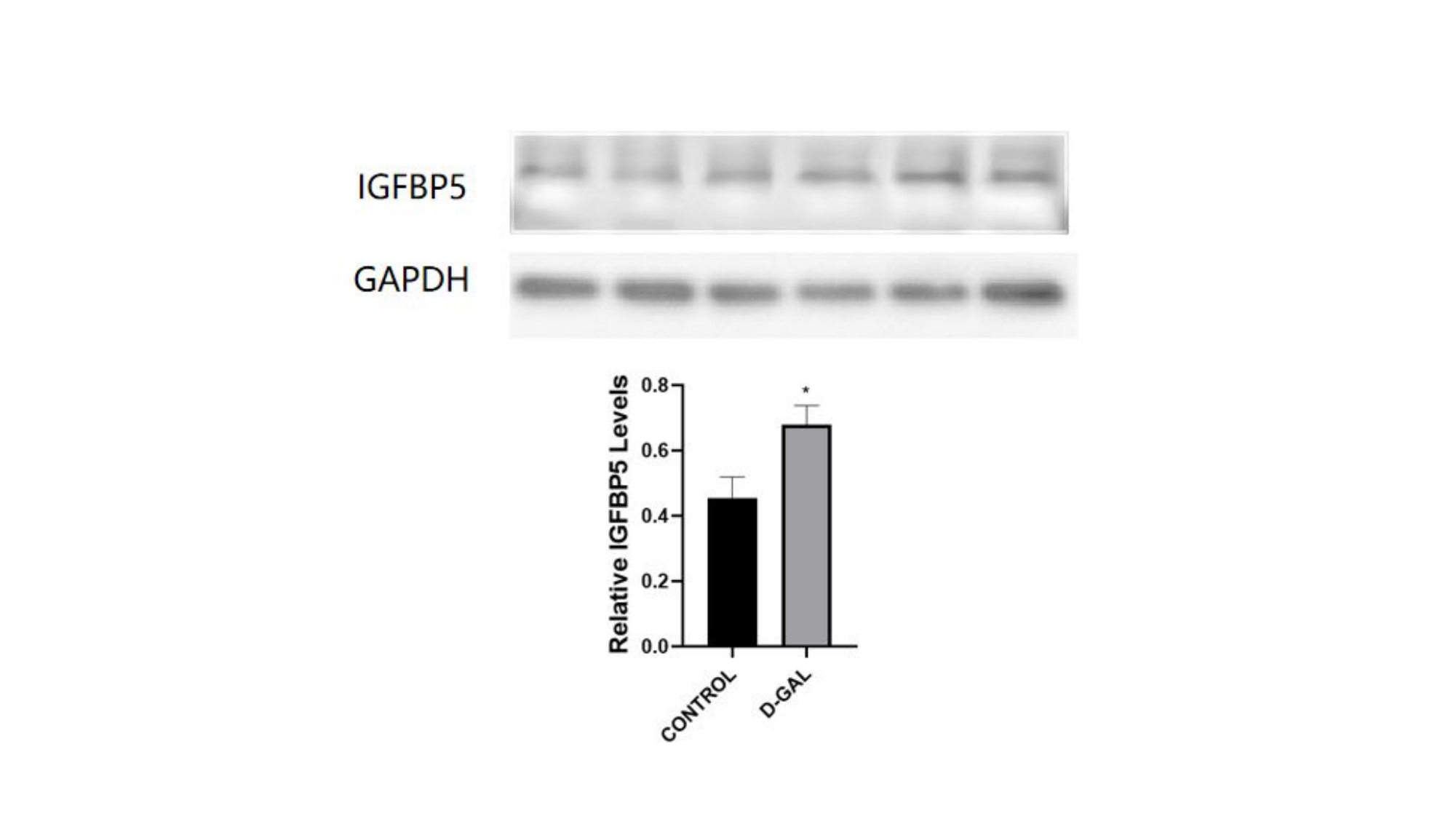

## Slide 3
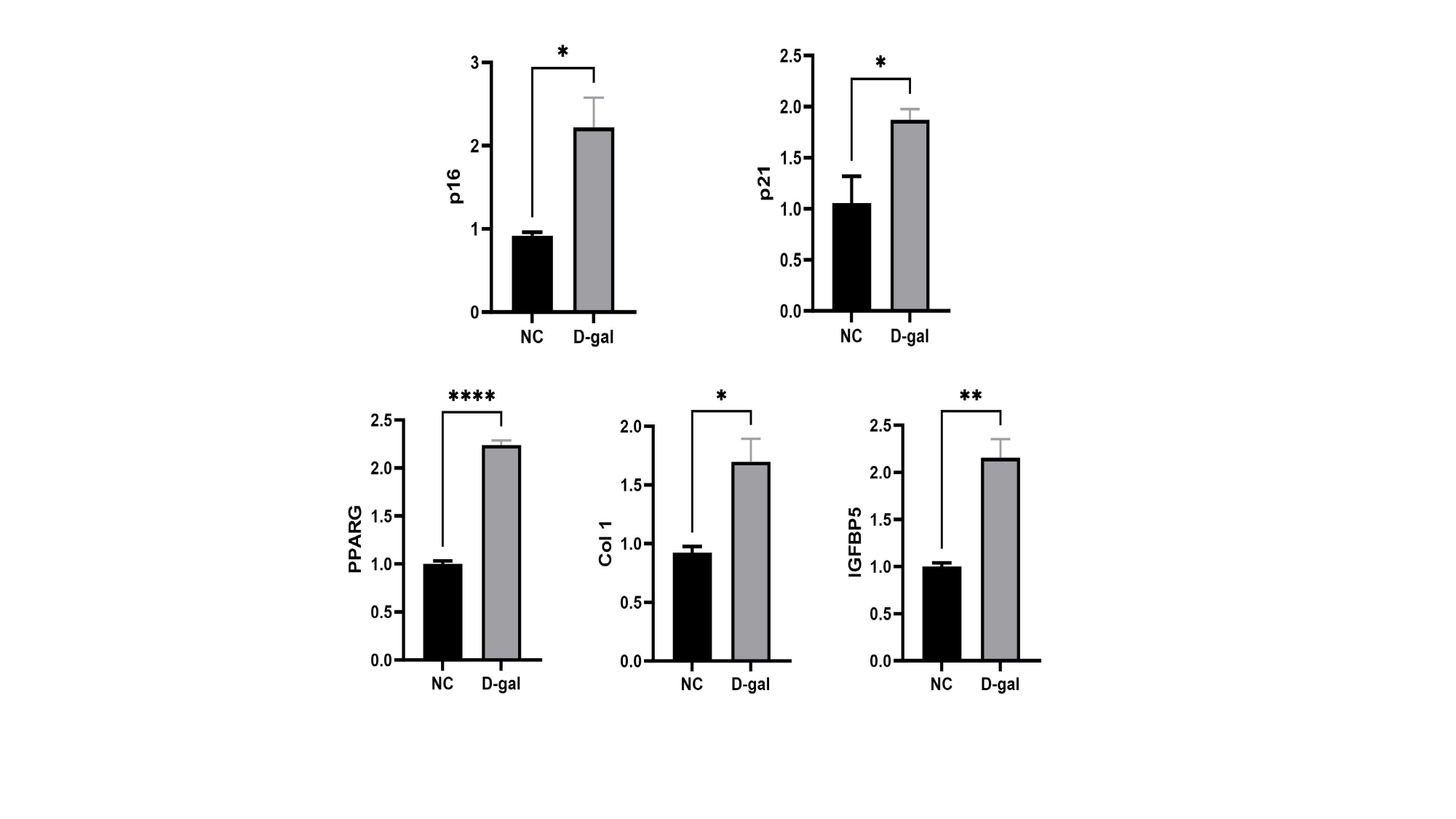

## Slide 4
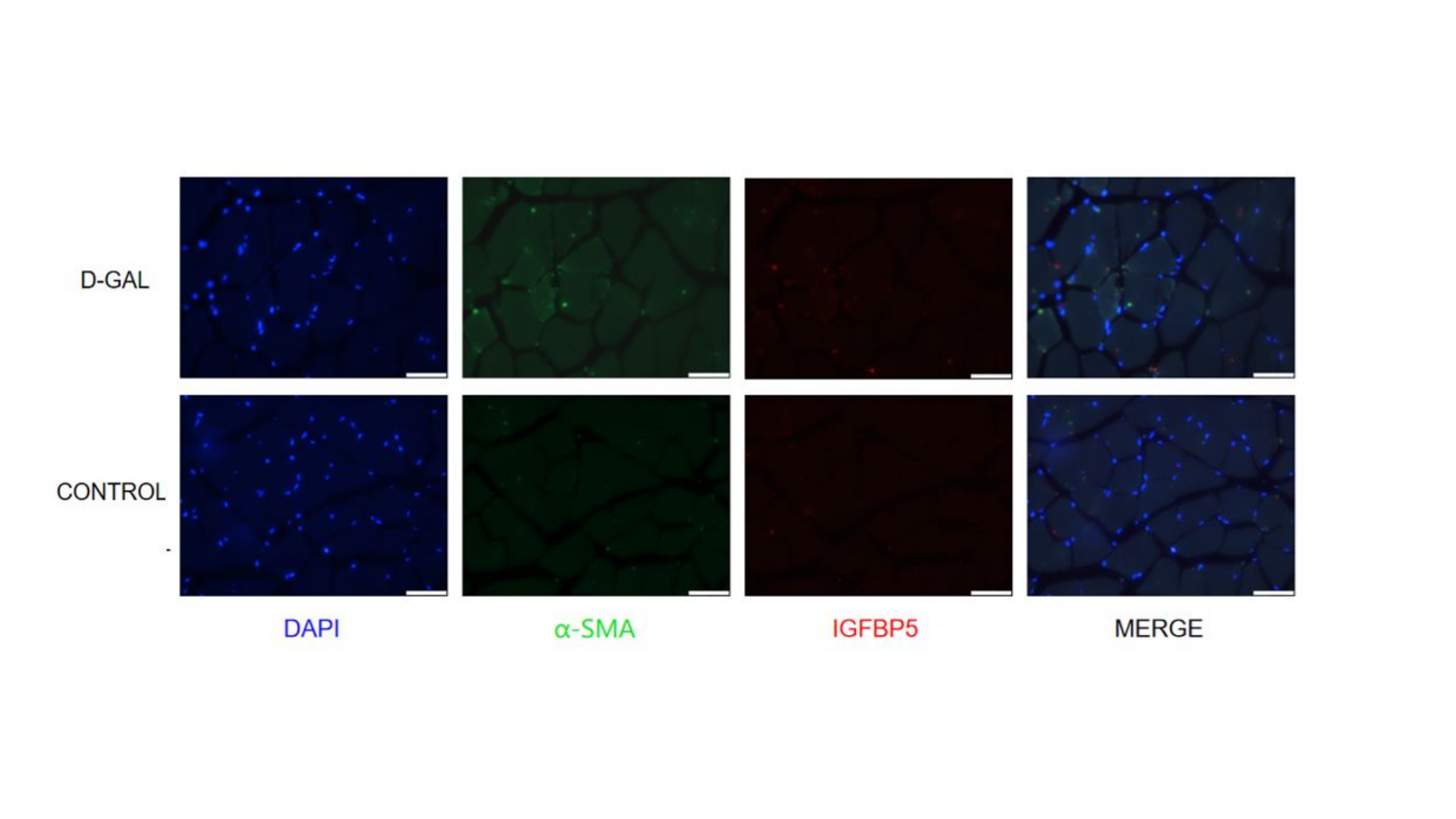

## Slide 5
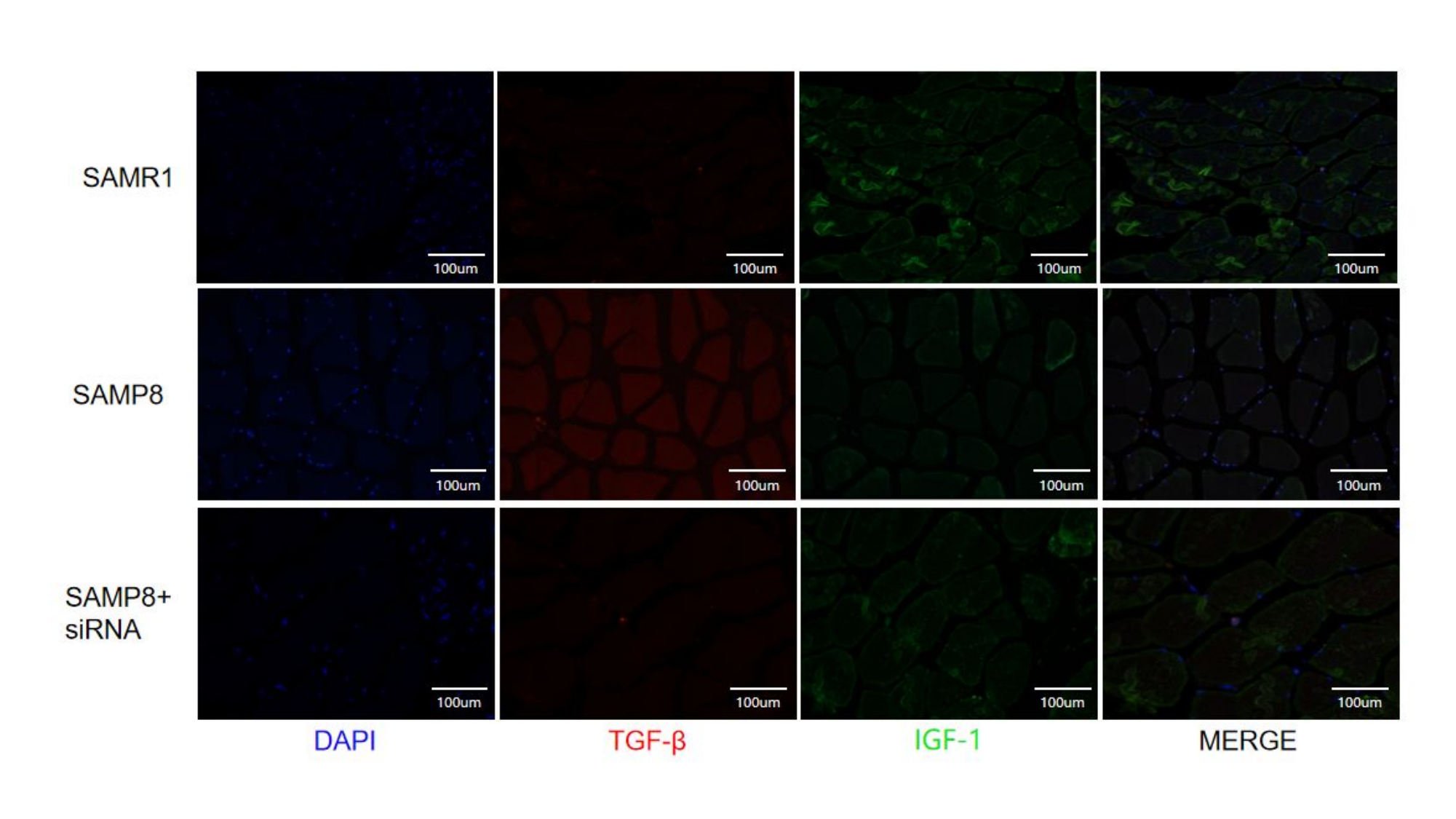

Supplement: Supplementary file 1 [file Presentation1.pptx]
