# Supplementary material for: Deciphering the role of IGFBP5 in delaying fibrosis and sarcopenia in aging skeletal muscle: therapeutic implications and molecular mechanisms
Source: Front Pharmacol. 2025 Mar 12;16:1557703. doi: 10.3389/fphar.2025.1557703 (PMC11937025; doi:10.3389/fphar.2025.1557703)

P16(FIGURE1 B)

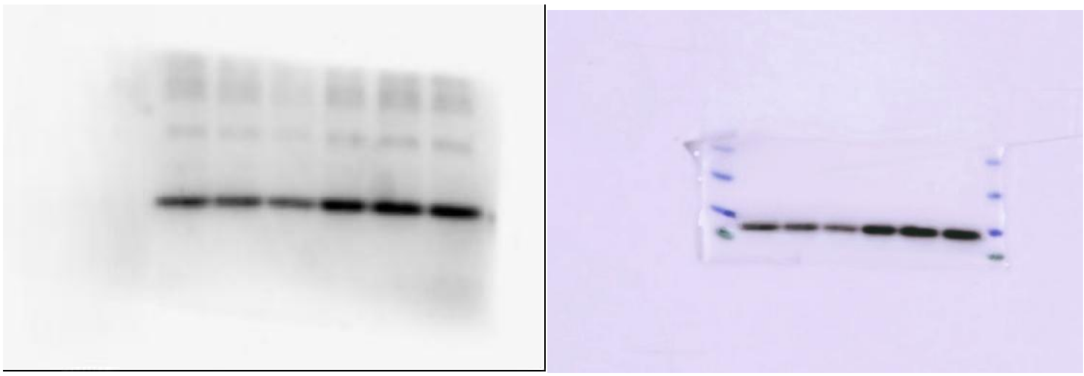

P53(FIGURE1 B)

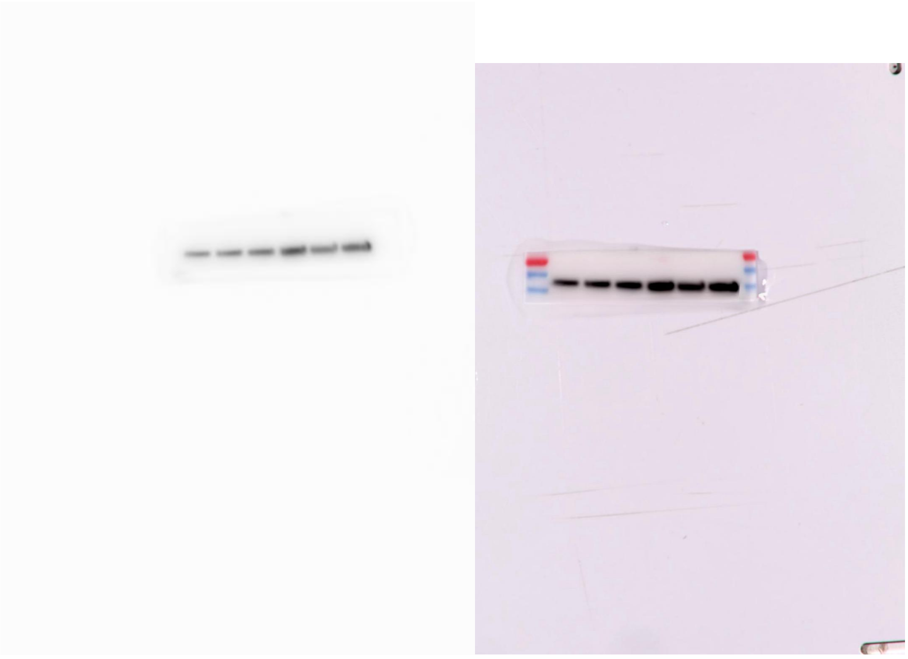

α -SMA(FIGURE1 C)

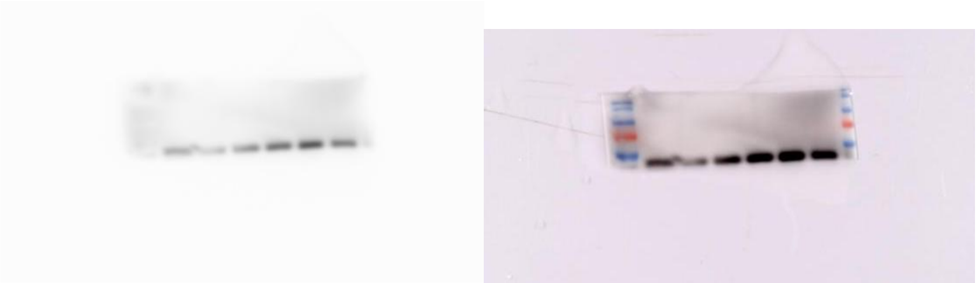

COL-1(FIGURE1 C)

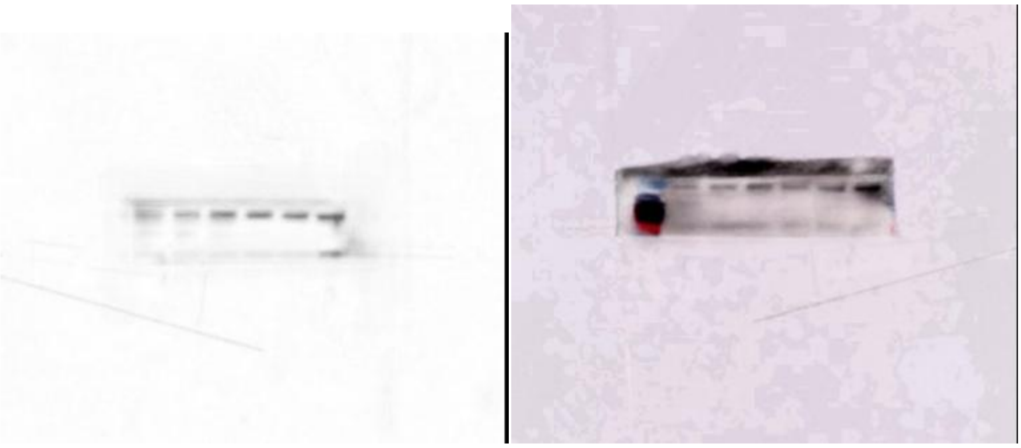

GAPDH(FIGURE1 B\C)---------All D-gal-induced samples(3 control group vs 3 D-gal-induced group) are the same.

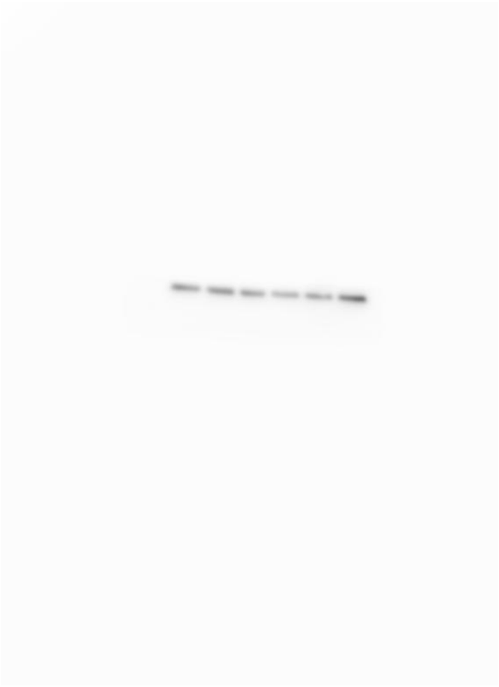

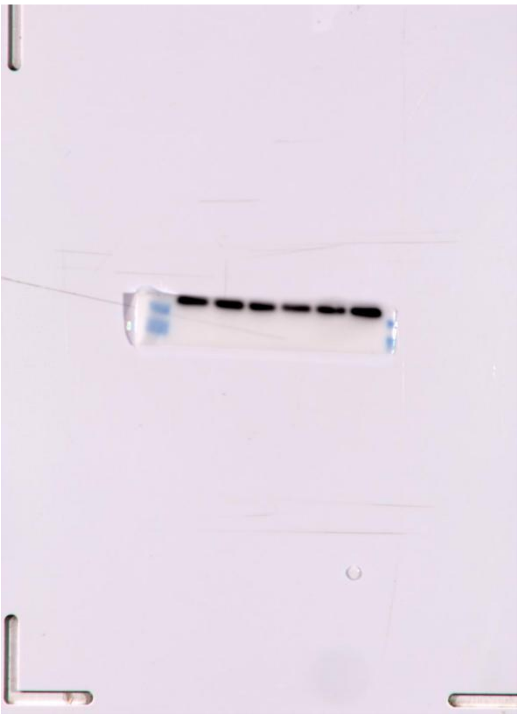

GAPDH(FIGURE 5C)

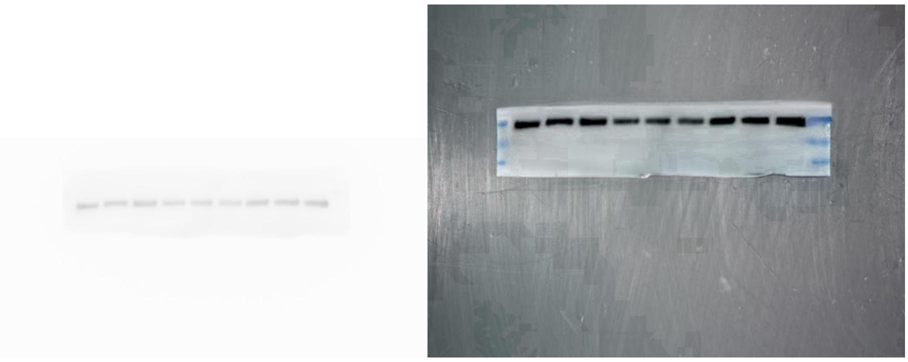

α -SMA(FIGURE 5C)

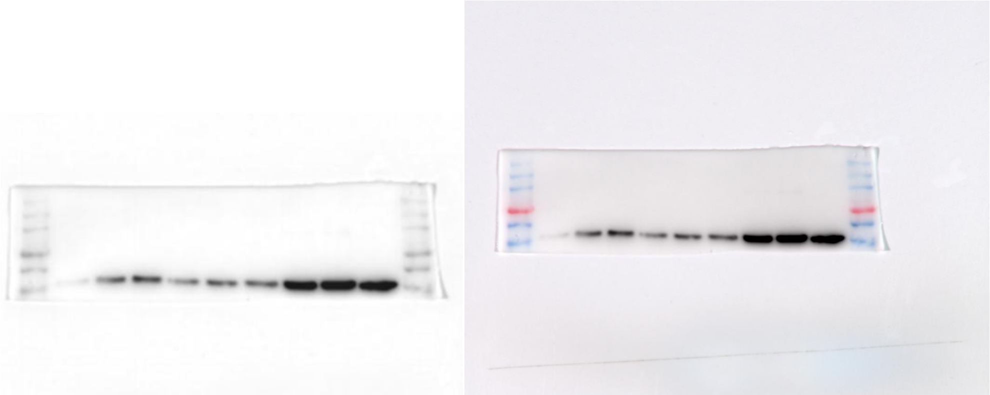

IGFBP5(supplymental image 2)

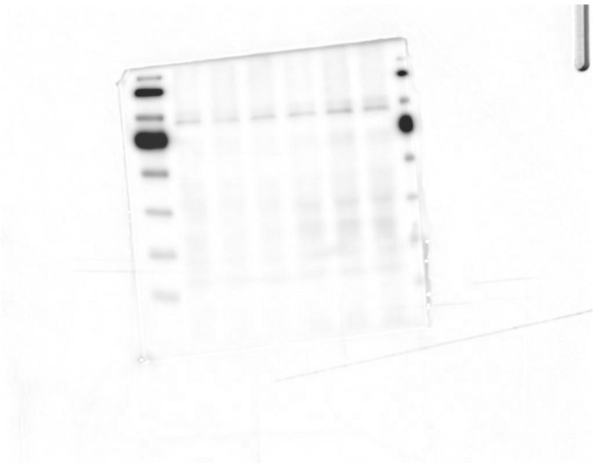

Supplement: Supplementary file 2 [file DataSheet1.pdf]
